# Supplementary material for: Chromosome segregation by the Escherichia coli Min system
Source: Mol Syst Biol. 2013 Sep 10;9:686. doi: 10.1038/msb.2013.44 (PMC3792344; doi:10.1038/msb.2013.44)
Supplement: Supplementary Information [file msb201344-s1.pdf]

Supplementary Information Table of contents

SI materials and methods	pages 1-14
References	pages 14-15
Supplementary Figures and Legends	pages 16-23
Supplementary Movies Legends	pages 24-25

Supporting information

SI Materials and Methods

Strains and plasmids

The strains and plasmids used in this study are listed in Supplementary Table 1. In order to purify His-tagged MinD, the *minD* coding sequence was cut out from plasmid pSR4 with restriction enzymes *Bam*HI/*Not*I and ligated into the vector pET28a, yielding plasmid pBDV-4. To purify the C-terminally truncated MinD$^{\Delta 10}$, a stop codon was introduced by site-directed mutagenesis in place of lysine 260 into plasmid pBDV-4, yielding plasmid pBDV-13. All point mutants were obtained by site-directed mutagenesis. EYFP-MinC was constructed by fusion PCR and cloned directly into plasmid pBAD33, yielding plasmid pBDV-15. The bi-cistronic construct MinD_MinE has been previously described (Di Ventura & Sourjik, 2011). This construct has been modified in this study by either mutating the *minD* gene (yielding plasmid pBDV-16) or by substituting *minD_minE-eyfp* with native *minD_minE* (yielding plasmid pBDV-17). This vector was then used as template for site-directed mutagenesis (yielding plasmid pBDV-18). The plasmid expressing SSB-YFP has been previously described (Thiem et al, 2007). We used this as a template to amplify by PCR *ssb-yfp*, which was subsequently cloned into pBAD33 using *Sac*I/*Hind*IIII restriction enzymes (yielding plasmid pBDV-21). To express native *minD* under an IPTG inducible promoter we amplified the gene by PCR using pSR4 as a template and cloned it back into plasmid pSR4 using *Nco*I/*Hind*III restriction enzymes (yielding plasmid pBDV-22).

Strain/ plasmid	**Relevant genotype**	**Reference**
MG1655	Wild-type; K-12 derivative	(Blattner et al, 1997)
MG1655 [*ΔminB*]	*ΔminB*::kan^R; MG1655 derivative	(Di Ventura & Sourjik, 2011)
PB114	*ΔminB*::kan^R; PB103 (*dadR trpE trpA tna*) derivative	(de Boer et al, 1989)
pBDV-4	MinD expression and purification plasmid; pET28a backbone; KAN resistance; IPTG inducible	This study
pBDV-5	MinD^{K11A} expression and purification plasmid; pET28a backbone; KAN resistance; IPTG inducible	This study
pBDV-6	MinD^{R187E} expression and purification plasmid; pET28a backbone; KAN resistance; IPTG inducible	This study
pBDV-7	MinD^{R219D} expression and purification plasmid; pET28a backbone; KAN resistance; IPTG inducible	This study
pBDV-8	MinD^{R251E} expression and purification plasmid; pET28a backbone; KAN resistance; IPTG inducible	This study
pBDV-9	MinD^{R254E} expression and purification plasmid; pET28a backbone; KAN resistance; IPTG inducible	This study
pBDV-10	MinD^{R251A_R254A} expression and purification plasmid; pET28a backbone; KAN resistance; IPTG inducible	This study
pBDV-11	MinD^{R251E_R254E} expression and purification plasmid; pET28a backbone; KAN resistance; IPTG inducible	This study
pBDV-12	MinD^{R92A_K94A_R151A_R155A} expression and purification plasmid; pET28a backbone; KAN resistance; IPTG inducible	This study
pBDV-13	MinD$^{\Delta 10}$ expression and purification plasmid; pET28a backbone; KAN resistance; IPTG inducible	This study
pBDV-14	MinD$^{R251E_R254E_\Delta 10}$ expression and purification plasmid; pET28a backbone; KAN resistance; IPTG inducible	This study
pBDV-15	EYFP-MinC expression plasmid; pBAD33 backbone; CAM resistance; Arabinose inducible	This study
pBDV-1	MinD_MinE-EYFP* expression plasmid; pTrc99a backbone; AMP resistance; IPTG inducible	(Di Ventura & Sourjik, 2011)
pBDV-16	MinD^{R219D}_MinE-EYFP* expression plasmid; pTrc99a backbone; AMP resistance; IPTG inducible	This study
pBDV-17	MinD_MinE* expression plasmid; pTrc99a backbone; AMP resistance; IPTG inducible	This study
pBDV-18	MinD^{R219D}_MinE* expression plasmid; pTrc99a backbone; AMP resistance; IPTG inducible	This study
pSR-4	EYFP-MinD expression plasmid; pTrc99a backbone; AMP resistance; IPTG inducible.	(Thiem et al, 2007)
pBDV-19	EYFP-MinD$_{R219D}$ expression plasmid; pTrc99a backbone; AMP resistance; IPTG inducible.	This study
pBDV-20	EYFP-MinD$_{R2E}$ expression plasmid; pTrc99a backbone; AMP resistance; IPTG inducible.	This study
pSR6	SSB-YFP expression plasmid; pTrc99a backbone; AMP resistance; IPTG inducible.	(Thiem et al, 2007)
pBDV-21	SSB-YFP expression plasmid; pBAD33 backbone; CAM resistance; Arabinose inducible.	This study
pBDV-22	MinD expression plasmid; pTrc99a backbone; AMP resistance; IPTG inducible.	This study

* Bi-cistronic constructs

Supplementary Table 1 Strains and plasmids used in this study.

Expression and purification of His-tagged wild-type and mutant MinD

Rosetta cells carrying the desired plasmid were grown to OD_{600} of 0.6 and induced with 1 mM IPTG for 3 hours at 37°C. Cells were collected by centrifugation (4.000 r.p.m.) at 4°C for 20 minutes and stored at -80°C. Pellets were thawed on ice and resuspended in lysis buffer (50 mM NaH_2PO_4 [pH 7.5], 300 mM NaCl, 10 mM imidazole, 10 mM β-mercaptoethanol, protease inhibitor [complete EDTA free, Roche Molecular Biochemicals]). After resuspension, cells were lysed by sonication and the crude lysate was clarified by centrifugation (20.000 r.p.m.) for 30 min at 4°C and subsequently filtered through a 0.2 mm filter. Proteins were purified using Profinia™ Protein Purification System (Bio-Rad), using the BIO-RAD® Bio-Scale™ Mini Profinity™ IMAC Cartridge 1mL and the BIO-RAD® Bio-Scale™ Mini Bio-Gel® P-6 Desalting Cartridge 10 mL. The Ni^{+2} column was washed with imidazole buffer (50 mM NaH_2PO_4 [pH 7.5], 300 mM NaCl, 20 mM imidazole, 10 % glycerol, 0.1 mM EDTA, protease inhibitor) and the protein was eluted with elution buffer (50 mM NaH_2PO_4 [pH 7.5], 300 mM NaCl, 250 mM imidazole, 10 % glycerol, 0.1 mM EDTA, protease inhibitor). The purification procedure was followed by exchange of elution buffer for storage buffer (50 mM HEPES [pH 7.25], 150 mM KCl, 10 % glycerol, 0.1 mM EDTA) and the purified proteins so obtained were stored in small aliquots at -80°C. Protein concentrations were estimated using a NanoDrop spectrophotometer (Thermo Scientific). Proteins were cleared from any aggregates by centrifugation (21.000 g) for 30 min at 4°C prior to use.

DNA Electrophoretic mobility shift assays (EMSA)

Binding reactions were performed in a volume of 10 μL in EMSA buffer (38 mM HEPES/NaOH [pH 7.2], 38 mM NaCl, 5 mM $MgCl_2$, 7 % glycerol, 1 mM DTT). Each reaction contained 200 fmol of dsDNA labelled by 5'-hexachloro-fluorescein phosphoramidite (HEX) and 1 mM ATP or ADP (unless specified otherwise). Reactions were incubated at room temperature for 10 min and then separated on 10% polyacrylamide (PA) native gel for ~30 min. Gels were run in 0.5 x TBE plus 1 mM $MgSO_4$ at 150 V and subsequently visualized using Typhoon gel scanner. All protein samples were adjusted to the same final concentration with the storage buffer (50 mM HEPES [pH 7.25], 150 mM KCl, 10 % glycerol, 0.1 mM EDTA) before loading on the gel, in order to ensure identical volumes in all lanes. DNA probes were either obtained by PCR (for the full-length P1 and pTrc promoters, 155 bp) or by annealing complementary oligonucleotides in a PCR cycler (for shorter fragments of the P1 or pTrc promoters).

Co-sedimentation of MinD with liposomes

Multilamellar large vesicles (320 μg/mL, obtained from *E. coli* total lipid extract, kind gift of Chris van der Does) were incubated for 10 min with wild type or mutant MinD (6 μM) and 1 mM of either ADP or ATP in 50 μL reaction volume of ATPase buffer (25 mM Tris-HCl [pH 7.5], 50 mM KCl, 5 mM $MgCl_2$, 5% glycerol). The reactions were pelleted by centrifugation at 21.000 g for 15-30 min, the pellets were resuspended in 50 μL ATPase buffer, and supernatant and pellet samples were analysed by SDS-PAGE, followed by Coomassie staining to detect MinD.

Co-sedimentation of DNA with MinD

Wild type or mutant MinD (1 μM) was incubated for 10 min with DNA (P1 promoter, 155 bp, 200 fmol) and 1 mM of either ADP or ATP in 25 μL reaction volume of the ATPase buffer (see above). The reactions were petted by centrifugation at 21.000 g for 15-30 min, and the pellets were resuspended in 25 μL ATPase buffer. Supernatant and pellet samples were analysed either on a native 10 % PA gel followed by visualization of the HEX-labelled DNA using a Typhoon™ Imager (GE Healthcare) or on the SDS-PAGE followed by Coomassie staining to detect the protein.

Flotation Assay

Liposome preparation for flotation assays

Lipid stock solution was prepared containing 3 mM synthetic DOPG (1,2-dioleoyl-*sn*-glycero-3-phospho-(1'-*rac*-glycerol) (sodium salt)) purchased from Avanti Polar Lipids and 0.1% DiO (3,3′-Dioctadecyloxacarbocyanine Perchlorate) from Invitrogen. The lipid mixture was initially dried under nitrogen to yield a homogeneous lipid film that was further dried under vacuum within an exciccator for 1.5h. Lipids were reconstituted in assay buffer (25 mM Tris-HCL pH 7.5, 50 mM KCL, 5 mM $MgCl_2$) to reach a final concentration of 4mM. Unilamellar liposomes were generated by 10 freeze/thaw cycles, following extrusion through a 400 nm filter (Mini-extruder; Avanti Polar Lipids) ending up with a diameter of 150 nm. The size of the small vesicles was determined by dynamic light scattering.

MinD Binding assay in flotation assay

In order to study protein interaction with both fluorescently labeled DOPG liposomes and DNA, these components were either together or separately incubated with 1 mM

ATP or ADP and recombinant wild type or mutant MinD (40µg/mL). Following incubation for 10 min at RT, protein binding was verified by floatation. For this purpose, total material was adjusted to 40% (w/v) Nycodenz at the bottom of a conical ultracentrifuge tube. The sample was covered with a layer of 30% (w/v) Nycodenz and assay buffer (25 mM Tris-HCl pH 7.5, 50 mM KCl, 5 mM $MgCl_2$) on top. The formed density gradient was centrifuged for 4 h at 48.000 RPM and 4°C prior to separation into four fractions. All material in the respective fractions has been recovered. Liposome associated material was mainly found in the upper fractions 1 and 2 (0/30% interface) while soluble material was present in lower fractions 3 and 4 (30/40% interface and 40% layer). All fractions were subjected to fluorescence measurements using a Gemini XS plate reader (Molecular Devices). Additionally, about 5% of each fraction was analyzed by SDS-PAGE and subsequent Western blotting using anti-polyHistidine antibodies. Quantification of protein amount was carried out by Licor infrared imaging system software.

DAPI staining

Cells were grown in medium A (1% Bacto-Tryptone, 0.5% NaCl, [pH 7]) at 34°C with appropriate antibiotics to mid-exponential phase, harvested by centrifugation (8.000 r.p.m., 1 min), washed and resuspended in a tethering buffer (10 mM potassium phosphate, 0.1 mM EDTA, 1 µM L-methionine and 10 mM sodium lactate [pH 7]), applied to microscopy slides pre-treated with poly-lysine and washed three times with water. 5 µL of DAPI (0.5 µg/mL in 50% glycerol) was added to the coverslip immediately before imaging.

Live-cell fluorescence microscopy

For live-cell imaging of the Min proteins, cells were grown to mid-exponential phase in LB at 34°C or 37°C with appropriate antibiotics. Protein expression was induced by isopropyl β-D-1-thiogalactopyranoside (IPTG) as specified in figure legends. For the analysis of SSB-YFP foci, MG1655*ΔminB* cells harbouring plasmids pSR6 and pTrc99a either empty or containing the *minD* gene were grown in M9 medium plus 0.4 % glucose until early exponential phase, induced with 5 µM IPTG for 1 h. Cells were harvested by centrifugation (8000 r.p.m., 1 min), washed, resuspended in tethering buffer (10 mM potassium phosphate, 0.1 mM EDTA, 1 µM L-methionine and 10 mM sodium lactate; pH 7) and applied to a thin agarose pad (0.5% agarose in tethering buffer, supplemented with 0.1% LB for time-lapse microscopy of growing cells). Fluorescence microscopy was performed at room temperature on a Zeiss AxioObserver.Z1 microscope equipped with an ORCA CCD camera (Hamamatsu). Images were analysed using ImageJ (http://rsbweb.nih.gov/ij/).

Automatic particle tracking and motion analysis

Mobility of SSB-YFP foci was analysed using automatic approach for tracking multiple fluorescent particles (Godinez et al, 2011; Godinez et al, 2009).

Briefly, for each time step of an image sequence, the tracking approach carries out four steps: 1) detection and localization of particles, 2) prediction of the position of the tracked particles, 3) matching of the predicted particle positions with the detected particle positions, and 4) position estimation. To detect and localize particles in a single image we used the spot-enhancing filter that is also known as Laplacian-of-Gaussian filter (Marr & Hildreth, 1980; Sage et al, 2005). The standard deviation $\sigma_{F,xy}$ of the filter is chosen based on the size of the particles (typically $\sigma_{F,xy}$ = 1.5 pixels).

Then a threshold for the filter response is applied and a connected-components labeling algorithm (8-connectivity) is used. The threshold for the filter response is automatically determined based on the mean intensity of the filtered image plus a factor c times the standard deviation. The position of each particle is determined by computing the intensity-weighted center of mass. To calculate a prediction for the position of each tracked particle, we used a Kalman filter with a Brownian motion model. For matching each predicted position with a detected particle position, we used a global nearest neighbour scheme (Sbalzarini & Koumoutsakos, 2005). Based on the predicted position as well as the detected particle position, the Kalman filter calculates a final position estimate for each tracked particle.

After tracking each individual particle, we calculated the mean value of the velocity (magnitude of the frame-to-frame displacement vector) over all available time steps of an individual particle. In addition, we determined the diffusion coefficient D of each particle. To calculate the diffusion coefficient D, we computed the mean square displacement (MSD) as a function of the time interval (Δt). We then fitted the standard diffusion model MSD (Δt) = $4D \cdot \Delta t$ (Saxton & Jacobson, 1997) to the calculated MSD values, thereby obtaining an estimate for D. To improve the robustness of the estimates and since we are mainly interested in the slope of the MSD curves, we only used the first five time intervals Δt. To determine the statistical significance of the differences in the D values from two different experiments, we used an independent two-sample t-test assuming unequal variances.

Surface Plasmon resonance analysis

Biosensor experiments were performed on a Biacore T100 system at 25°C and a flow rate of 30 µL/min in buffer A (50 mM HEPES pH 7.2, 150 mM KCl, 0.1 mM EDTA,

5% glycerol, 5 mM $MgCl_2$, 0.005% Tween 20). The biotinylated double-stranded oligonucleotides (5' sequence: P1-prom-1-BiotinF: GAATCAGCGCCATTTATCACAGAATAGACT) were immobilized on a streptavidin-coated Sensor Chip SA (GE Healthcare) according to the manufacturer's recommendations. After each binding reaction, the chip surface was regenerated washing for 20 s with 7 mM NaOH. In all experiments, a reference cell lacking DNA was used to detect the non-specific binding of the proteins to the Sensor Chip, which was then subtracted from all the obtained binding curves.

Estimating the rates of binding (*k_{on}*) and unbinding (*k_{off}*) of MinD to DNA using surface plasmon resonance (SPR) experiments

In order to estimate k_{on} and k_{off} of MinD-DNA binding, we used the 3 curves obtained with SPR at the highest MinD concentrations: 1, 2.5 and 5 μM.

We assumed a sequential binding mode of MinD to DNA:

$$A + B \underset{k_{-1}}{\overset{k_1}{\leftrightarrow}} A \cdot B \underset{k_{-2}}{\overset{k_2}{\leftrightarrow}} (A \cdot B)^*$$

$$\begin{aligned} k_1 &= m_1 \\ k_{-1} &= t_1 - \frac{m_2}{m_1} \\ k_{-2} &= \frac{t_2}{t_1 - \frac{m_2}{m_1}} \\ k_2 &= \frac{m_2}{m_1} - \frac{t_2}{t_1 - \frac{m_2}{m_1}} \end{aligned} \qquad (1)$$

To obtain the values of m_1, m_2, t_1 and t_2 we first needed to fit the ascending (binding) part of each curve to two exponentials:

$$Y_{max1}\ (1-e^{-\lambda_1 x}) + Y_{max2}\ (1-e^{-\lambda_2 x})$$

to find λ_1 and λ_2 (λ_1 faster rate, λ_2 slower rate).

By plotting $\lambda_1 + \lambda_2$ and $\lambda_1 \cdot \lambda_2$ versus [A] (that is, versus MinD concentration) and fitting the points to the equation of a line: $m_i x + t_i$, we could find the values of m_1, m_2, t_1 and t_2 using the values of the slope m and the intercept t.

Plugging these values into the equations in (1), we found the following k_{on} and k_{off}:

k_{on1} = 2.874 10^6 M

k_{off1} = 17.19991 sec^{-1}

k_{on2} = 0.03860 10^6 M

k_{off2} = 0.021488484 sec^{-1}

The descending (unbinding) part of the SPR curves can also be used to estimate k_{off} and we should be able to find values similar to those found using the ascending part of the curve. We therefore used the cftool of Matlab to fit the descending curves corresponding to MinD concentrations of 1, 2.5 and 5 µM to two exponentials:

$$a e^{bx} + c e^{dx}$$

Averaging the 3 values obtained for *b* and *d*, we get:

<*b*> = 0.02244 sec^{-1}

<*d*> = 0.002228 sec^{-1}

<*d*> is likely to represent the unspecific binding of MinD to the CHIP surface. <*b*> is instead in excellent agreement with the value we obtained when using the binding part of the curve (k_{off2} = 0.021488484 sec^{-1}).

k_{off1} = 17.19991 sec^{-1} obtained when using the binding part of the curve is likely to represent a fast unbinding that can be seen in the very first part of the descending SPR curve, but due to lack of sufficient time points, our fitting is not able to find this rate as well.

We conclude that the relevant k_{off} of MinD-DNA interaction is given by <*b*> = 0.02244 sec^{-1}

This leads to a dwell time of:

$$\text{dwell} = 1/k_{off} = \sim 44 \text{ sec}$$

Modelling and simulation of chromosome dynamics with and without the Min system

Two *E. coli* sister chromosomes were described as two self-avoiding ring polymers (Fritsche et al, 2012) that can move in a geometry that approximates a bacterial cell – an elongated rectangular parallelepiped of aspect ratio 1:8. For polymer rings of lengths $N = 80$, the linear dimensions of the confining geometry were set up such that

the radius of gyration R^{free}_{gyr} of the unconfined chain is larger than the linear square box sizes, leading to a 80 × 10 × 10 lattice size and volume fraction of a single chain of 10 %. Overlapping configurations of two chains, whose centers of mass coincide with the middle of the cell's long axis, were created to initiate the segregation process. Independent Monte Carlo trajectories (different initial conditions driven by different random number sequences) representing the dynamics of the segregation process were then sampled.

In order to study the impact of non-specific DNA tethering to the membrane and to mimic the oscillatory behaviour of the MinD protein, we performed simulations representing the following biological situations: (i) mutant case without possibility of tethering, (ii) homogeneous tethering probability, (iii) fixed gradient of tethering probability, and (iv) oscillating tethering probability (representing MinD oscillations). Tethering is implemented by temporarily fixing monomers that approach the border of the confinement, with a probability ρ proportional to the density of tethering sites at that position. A tethered monomer dissociates from the tethering site after a fixed time, set to 10 MCS in our simulations. If it stays in the vicinity of the membrane it can rebind immediately, with a probability given by the current density of tethering sites at this location. The tethering probability is set to $\rho = 0$ for case (i), and has the same average value of $\rho = 0.25$ for cases (ii)-(iv).

The static gradient (iii) is V-shaped, given by

$$\rho_{\text{grad}}(x) = \begin{cases} -\frac{x}{L} + \frac{1}{2} & \text{if } x \leq \frac{L}{2}, \\ \frac{x}{L} - \frac{1}{2} & \text{if } x > \frac{L}{2}, \end{cases}$$

where x is the position along the long axis of the cell and L is the length of the cell in that direction (Supplementary Figure 1B). The oscillating distribution consists of two linear gradients that span from the left of the cell decreasing to zero at a distance d(t), and from the right of the cell decreasing to zero at a distance L/2 – d(t) (Supplementary Figure 1C).

More precisely, it is given by

$$\rho_{\text{oscill}}(x,t) = \begin{cases} -\frac{x}{d(t)} + 1 & \text{if } x < d(t), \\ 0 & \text{if } d(t) \leq x \leq \frac{L}{2} + d(t), \\ \frac{1}{\frac{L}{2} - d(t)} \left[x - \left(\frac{L}{2} + d(t)\right)\right] & \text{if } \frac{L}{2} + d(t) < x, \end{cases}$$

where:

$d(t) = \frac{L}{2} \cos\left(\pi \frac{t}{T}\right)^2$

The period T is chosen so that there are approximately 10 oscillations during the segregation process.

The Bond-Fluctuation Method

We employ the bond-fluctuation method (BFM) (Carmesin & Kremer, 1988), which has been applied successfully to model the static and dynamic properties of polymer systems in several investigations (Binder & Heermann, 2002). It is a coarse-grained lattice model with the advantage of avoiding non-ergodicity, and its computational efficiency renders it more attractive than off-lattice models.

Each monomer occupies a unit cube of 8 sites on the lattice and is connected by a pair of bonds, where the length of a bond can take the values 2, 3, √5, √6 and √10, therewith leading to an average bond length of < b > = 2.7. The simulation method

produces unbiased results, takes into account excluded volume interactions and ensures that no bond crossings can occur (*i.e.* the topology is preserved).

References

Binder K, Heermann DW (2002) Monte Carlo Simulation in Statistical Physics: An Introduction. *Springer series in solid-state sciences* **80**

Blattner FR, Plunkett G, 3rd, Bloch CA, Perna NT, Burland V, Riley M, Collado-Vides J, Glasner JD, Rode CK, Mayhew GF, Gregor J, Davis NW, Kirkpatrick HA, Goeden MA, Rose DJ, Mau B, Shao Y (1997) The complete genome sequence of Escherichia coli K-12. *Science* **277:** 1453-1462

Carmesin I, Kremer K (1988) The bond fluctuation method: a new effective algorithm for the dynamics of polymers in all spatial dimensions. *Macromol* **21:** 2819

de Boer PA, Crossley RE, Rothfield LI (1989) A division inhibitor and a topological specificity factor coded for by the minicell locus determine proper placement of the division septum in E. coli. *Cell* **56:** 641-649

Di Ventura B, Sourjik V (2011) Self-organized partitioning of dynamically localized proteins in bacterial cell division. *Mol Syst Biol* **7:** 457

Fritsche M, Li S, Heermann DW, Wiggins PA (2012) A model for Escherichia coli chromosome packaging supports transcription factor-induced DNA domain formation. *Nucleic Acids Res* **40:** 972-980

Godinez WJ, Lampe M, Eils R, Müller B, Rohr K (2011) Tracking multiple particles in fluorescence microscopy images via probabilistic data association. *Proc 8th IEEE Internat Symposium on Biomedical Imaging: From Nano to Macro (ISBI'11)***:** 1925 – 1928

Godinez WJ, Lampe M, Worz S, Muller B, Eils R, Rohr K (2009) Deterministic and probabilistic approaches for tracking virus particles in time-lapse fluorescence microscopy image sequences. *Med Image Anal* **13:** 325-342

Marr D, Hildreth E (1980) Theory of edge detection. *Proc R Soc Lond B Biol Sci* **207:** 187-217

Sage D, Neumann FR, Hediger F, Gasser SM, Unser M (2005) Automatic tracking of individual fluorescence particles: application to the study of chromosome dynamics. *IEEE transactions on image processing : a publication of the IEEE Signal Processing Society* **14:** 1372-1383

Saxton MJ, Jacobson K (1997) Single-particle tracking: applications to membrane dynamics. *Annual review of biophysics and biomolecular structure* **26:** 373-399

Sbalzarini IF, Koumoutsakos P (2005) Feature point tracking and trajectory analysis for video imaging in cell biology. *J Struct Biol* **151:** 182-195

Thiem S, Kentner D, Sourjik V (2007) Positioning of chemosensory clusters in E. coli and its relation to cell division. *EMBO J* **26:** 1615-1623

**Mutated residue(s)**	**Effect on DNA binding**[a]	**Effect on membrane binding**	
		In vitro[b]	***In vivo***[c]
K11A	Unaffected	Abolished	N.A.
R187E	Unaffected	N.A.[d]	N.A.
R219D	Strongly impaired	Impaired	Impaired
R251E	Unaffected	N.A.	N.A.
R254E	Unaffected	N.A.	N.A.
R251A_R254A	Unaffected	N.A.	N.A.
R251E_R254E (R2E)	Unaffected	Impaired	Impaired
Δ10	Nearly abolished	Abolished	N.A.
R2EΔ10	Unaffected	N.A.	N.A.
R92E_K94E_R151E_R155E	Unaffected	N.A.	N.A.

[a]Analysed using EMSA.

[b]Analysed using MinD-liposomes co-sedimentation assay.

[c]Analysed using fluorescent microscopy with YFP-labeled MinD.

[d]N.A., not analysed.

Supplementary Table 2 Membrane and DNA binding by MinD mutants tested in this study.

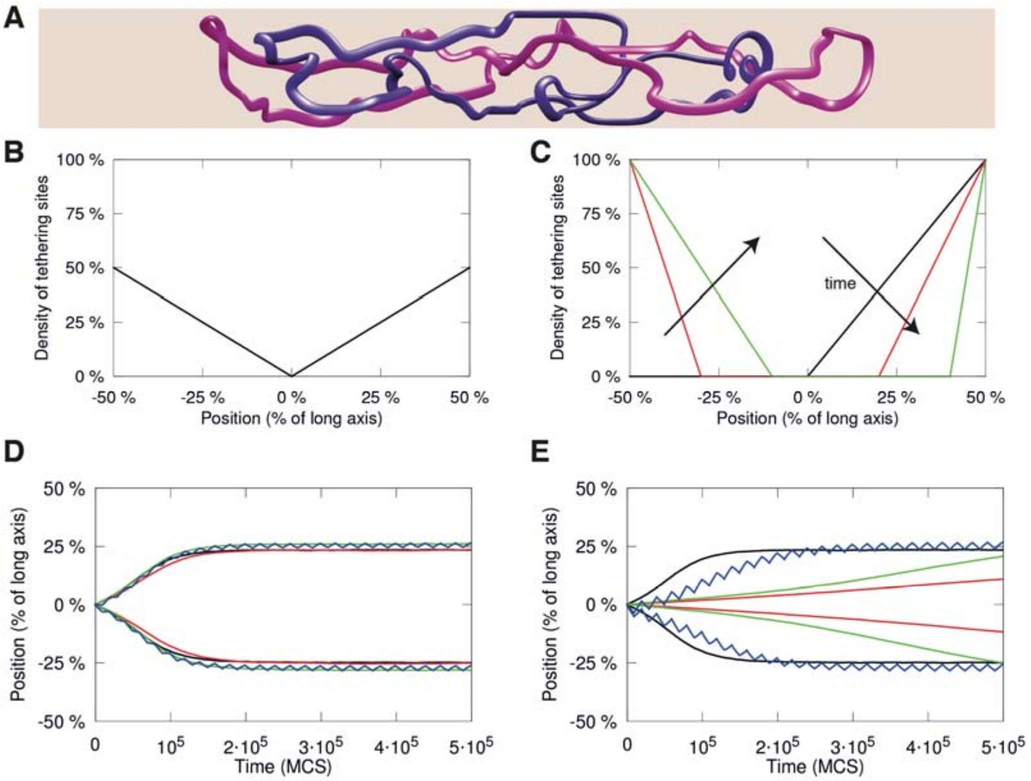

Supplementary Figure 1 Simulating chromosome segregation by a gradient of DNA tethering sites on the membrane. (**A**) Example of initial position of sister chromosomes used in the simulations. The box represents the elongated rectangular parallelepiped of aspect ratio 1:8 that was used to approximate an *E. coli* cell shape. Chromosomes were simulated as two self-avoiding ring polymers. (**B**) Shape of the static gradient of DNA tethering sites used in the simulations. (**C**) Shape and movement of the oscillating gradient of DNA tethering sites used in the simulations. First time point, blue. Second time point, orange. Third time point, pink. (**D**) Position of the center of mass (CM) of each polymer relative to the long cell axis as a function of time, measured in Monte-Carlo steps (MCS), when using half the density of tethering sites used in the simulations shown in Figure 1A. (**E**) Same as in (D), but using a dwell time of DNA segments at the tethering sites that is 10 times longer than the one used in Figure 1A.

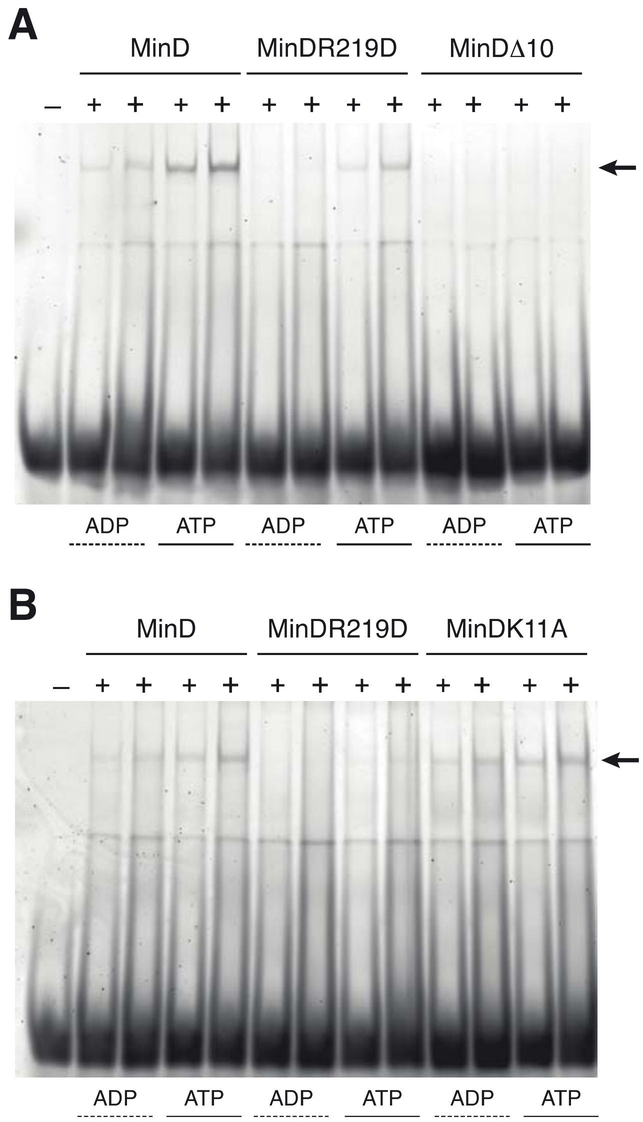

Supplementary Figure 2 Electrophoretic mobility shift assays (EMSA) with MinD mutants. (**A-B**) Fluorescently labeled DNA (HEX-pTrc, 28 bp, 200 fmol) was incubated with wild-type and indicated mutant MinD proteins, samples were separated on native 6 % polyacrylamide gel and DNA was detected using fluorescence measurements. The minus symbol indicates the control samples containing only the DNA probe. Black arrows indicate nucleoprotein complexes. Increasing protein amounts, indicated by plus symbols of increasing size, are 0.86 µM and 3.2 µM, respectively. ADP or ATP was used, when indicated, at a final concentration of 1 mM.

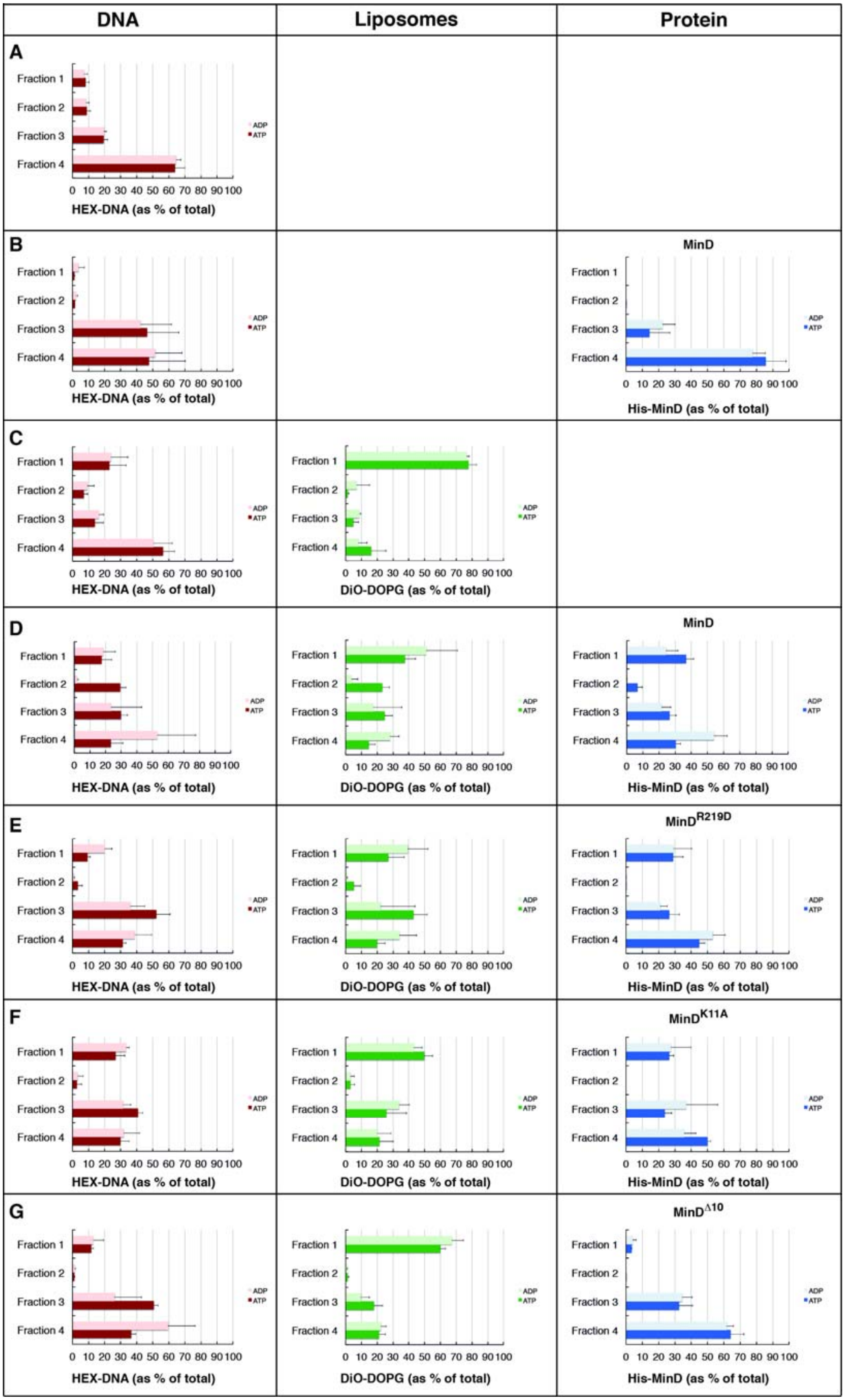
DNA
Liposomes
Protein
A
B
C
D
E
F
G
MinD
MinD^{R219D}
MinD^{K11A}
MinD$^{\Delta 10}$
Fraction 1
Fraction 2
Fraction 3
Fraction 4
ADP
ATP
0 10 20 30 40 50 60 70 80 90 100
HEX-DNA (as % of total)
DiO-DOPG (as % of total)
His-MinD (as % of total)

Supplementary Figure 3 Flotation experiments with DOPG liposomes, DNA and MinD. Binding and flotation behaviour were analyzed by applying density gradients for fluorescently labelled liposomes and DNA (see Methods), either alone or combined with wild-type or mutant MinD proteins. ADP or ATP was used in the reactions at a final concentration of 1 mM. All fractions were subjected to fluorescence measurements to detect liposomes and DNA and to immunoblotting with anti-polyHistidine antibodies to detect MinD. (**A**) Flotation of HEX-labelled DNA (P1 promoter, 155 bp, 200 fmol) alone. (**B**) Flotation of HEX-labelled DNA (P1 promoter, 155 bp, 200 fmol) together with His-MinD (1.3 μM). (**C**) Flotation of DOPG liposomes (400μg/ml) with HEX-DNA. (**D-G**) Flotation of the indicated protein (1.3 μM) with liposomes and HEX-DNA.

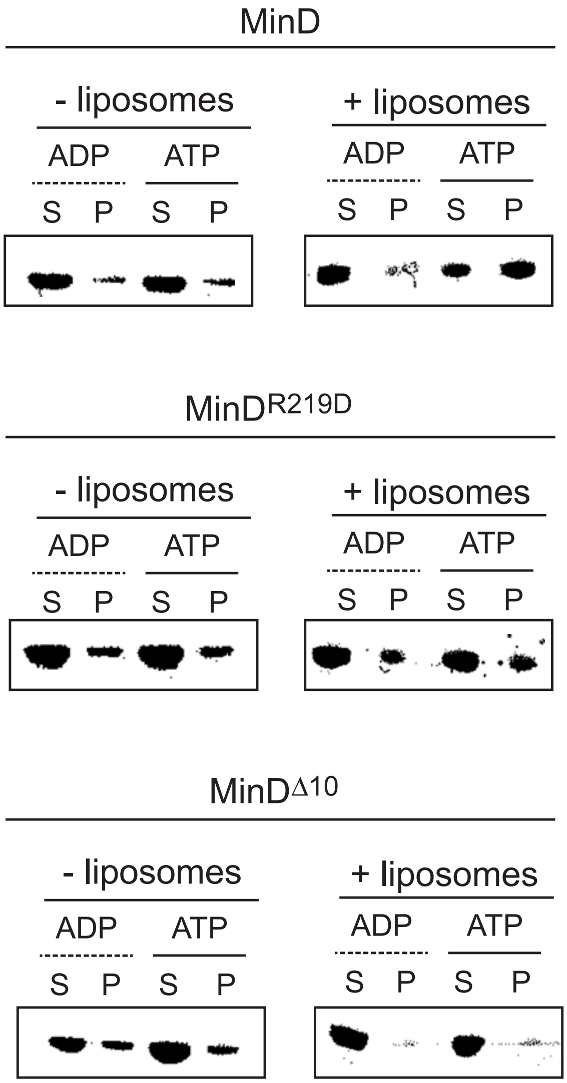

Supplementary Figure 4 Co-sedimentation assay of MinD with liposomes. 1 µM protein (wild type or mutant, as indicated) was incubated without or with liposomes (320 µg/mL) and either ADP and ATP (1 mM). Reactions were incubated for 10 min at RT and then centrifuged at 21.000 g for 30 min. Pellets were resuspended in ATPase buffer and supernatants and pellets were analysed by SDS-PAGE, visualised by Coomassie staining. Since the pellet fraction represents material that is bound to the liposomes, the presence of protein in this fraction is indicative of binding.

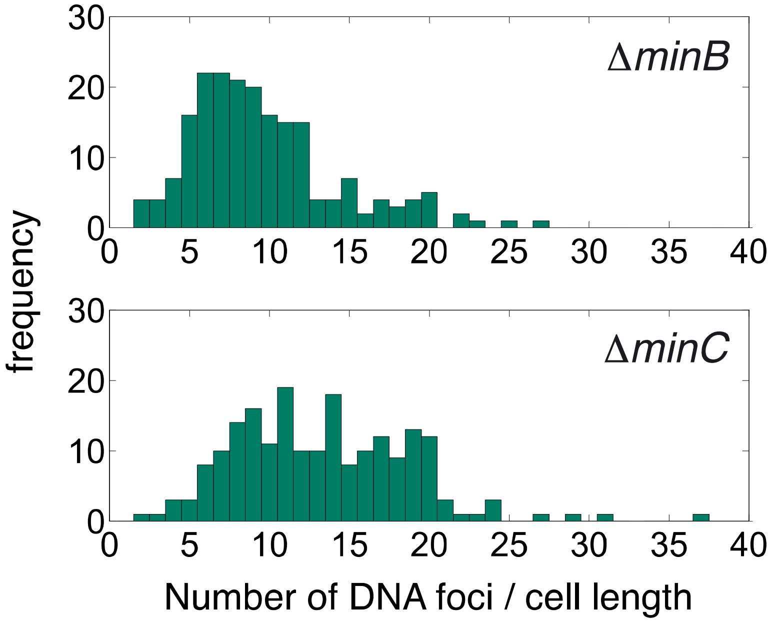

Supplementary Figure 5 Absence of MinD leads to chromosome segregation defects *in vivo*. Histograms showing the distribution of the number of distinct nucleoids divided by cell length over a population of 200 cells for *ΔminB* (upper panel) and *ΔminC* (lower panel) cells.

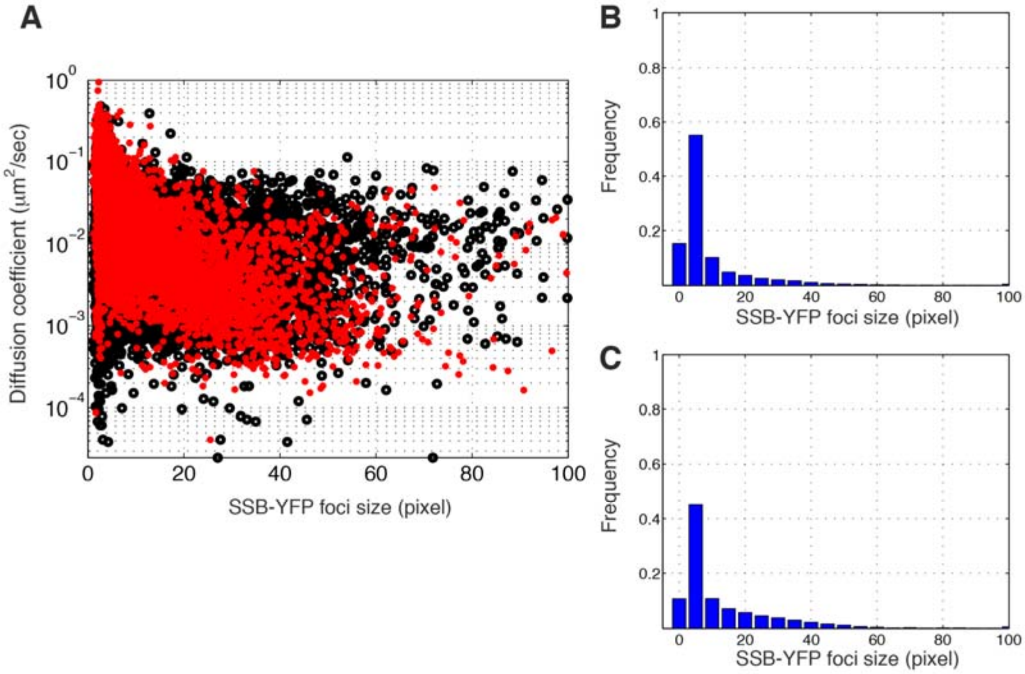

Supplementary Figure 6 *ΔminB* cells expressing an empty plasmid or MinD show a similar distribution of SSB-YFP foci size. 0.01% arabinose (to express SSB-YFP) and 10 μM IPTG (to express MinD) were added to both strains for 4 h. (**A**) Diffusion coefficient plotted versus SSB-YFP foci size (calculated in pixels) for cells expressing an empty plasmid (red) or MinD (black). (**B-C**) Histograms showing the distribution of SSB-YFP foci size in the absence (B) or presence (C) of MinD.

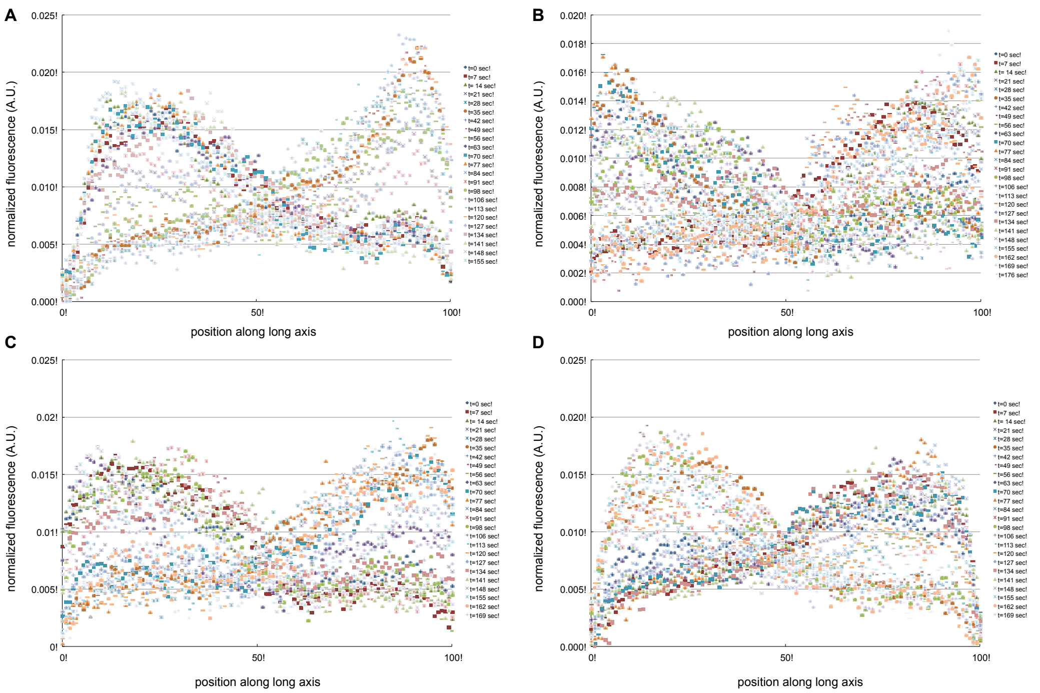

Supplementary Figure 7 Spatial profiles of EYFP-MinD for selected time points of the Min oscillation cycle. EYFP-MinD was expressed in MG1655 cells at 30 μM IPTG induction and imaged for several minutes every 7 seconds. The mean fluorescence intensity profile was then plotted along the long cell axis at each time point for several oscillation cycles. (**A-D**) MinD profiles in four individual cells are shown. The mean fluorescence appears to go down at the cell ends because the box along which the fluorescence profile was plotted was slightly longer than the cell itself.

Supplementary Movie 1

Representative time-lapse movie of *ΔminB* cells expressing MinD_MinE-EYFP from a bi-cistronic construct. Cells were grown at 34°C in LB medium with 30 μM IPTG for four hours. At these high induction levels, most cells show aberrant MinE-EYFP localization, with only few cells displaying the expected formation of dynamic MinE ring. Images were recorded every 15 seconds. The movie runs at 3 frames per second.

Supplementary Movie 2

Representative time-lapse movie of *ΔminB* cells expressing EYFP-MinC and MinD/MinE. Cells were grown at 34°C in LB medium with 0.01% arabinose (to induce EYFP-MinC) and 30 μM IPTG (to induce untagged MinD_MinE from a bi-cistronic construct) for four hours. Due to the high induction level of MinD/MinE, EYFP-MinC localization is aberrant compared to low induction levels (see Supplementary Movie 5). Images were recorded every 15 seconds. The movie runs at 3 frames per second.

Supplementary Movie 3

Same as in Supplementary Movie 1, but with the $MinD^{R219D}$ instead of the wild-type MinD.

Supplementary Movie 4

Same as in Supplementary Movie 2, but with the $MinD^{R219D}$ instead of the wild-type MinD.

Supplementary Movie 5

Same as in Supplementary Movie 2, but with cells grown in presence of 10 μM IPTG to induce MinD/MinE.
